# Comparative Study of ZnO and ZnO-Ag Particle Synthesis via Flame and Spray Pyrolysis for the Degradation of Methylene Blue

**DOI:** 10.3390/molecules30163364

**Published:** 2025-08-13

**Authors:** Kusdianto, Nurdiana Ratna Puri, Adhi Setiawan, Sugeng Winardi, Widiyastuti, Suci Madhania, Mohammad Irwan Fatkhur Rozy, Manabu Shimada

**Affiliations:** 1 Chemical Engineering Department, Institut Teknologi Sepuluh Nopember, Kampus ITS Sukolilo, Surabaya 60111, Indonesia; 7008232003@student.its.ac.id (N.R.P.); adhi.setiawan@ppns.ac.id (A.S.); swinardi@chem-eng.its.ac.id (S.W.); widi@its.ac.id (W.); suci@its.ac.id (S.M.); irwan@its.ac.id (M.I.F.R.); 2 Waste Treatment Engineering Study Program, Shipbuilding Institute of Polytechnic Surabaya, Jalan Teknik Kimia, Kampus ITS Sukolilo, Surabaya 60111, Indonesia; 3 Chemical Engineering Program, Graduate School of Advanced Science and Engineering, Hiroshima University, 4-1, Kagamiyama 1-Chome, Higashi-Hiroshima, Hiroshima 739-8527, Japan; smd@hiroshima-u.ac.jp

**Keywords:** gas phase, composite, degradation efficiency, photocatalytic

## Abstract

The treatment of organic waste from dyes or other industry processes is a crucial issue that requires urgent attention. Photocatalysis is a promising method for tackling this problem, with ZnO being a commonly used photocatalyst material. This study compared the degrading efficiency of ZnO particles and ZnO-Ag composites by utilizing flame and spray pyrolysis techniques. Under UV light, methylene blue (MB) was used as a model organic waste. The generated particles were characterized using Brunauer–Emmett–Teller (BET) surface area, scanning electron microscopy (SEM), X-Ray diffraction (XRD), and a UV-Vis spectrometer. The findings showed that the ZnO and ZnO-Ag obtained using both methods exhibited hexagonal Wurtzite crystal structures, and there was no significant difference in the crystal sizes produced. SEM analysis indicated that the morphology of the resulting particles differed significantly, with flame-synthesized particles being remarkably smaller in size (one-thirtieth the size following spray synthesis) and having smoother surfaces. Furthermore, the addition of Ag particles to ZnO enhanced the MB degradation efficiency by two to three times, achieving a maximum of 64% at 75 min. The BET analysis showed that the surface area of ZnO doped with Ag was larger compared to that of pristine ZnO. On the other hand, the ZnO-Ag particles produced via spray pyrolysis exhibited a total pore volume (determined through nitrogen adsorption–desorption analysis) three times larger than that of the particles produced via the flame method. The particles produced via spray pyrolysis also had better MB degradation performance compared to those synthesized using flame pyrolysis.

## 1. Introduction

Indonesia is among the largest textile-exporting countries in the world. However, the increasing textile production is contributing to a rise in the amount of organic dye waste generated. Besides organic waste from textile dyes, household industries, paints, and food products are also potential sources of organic waste. Several methods are used for organic waste degradation, including adsorption, membrane filtration, oxidation, biodegradation, and photocatalysis [1,2,3,4,5]. Photocatalytic methods are considered highly promising for waste degradation due to their speed and relatively high degradation rate. Several semiconductor materials can be used as photocatalyst materials, including ZnO, TiO_2_, MnO_2_, CeO_2_, and SnO_2_ [5,6,7,8,9,10]. ZnO is extensively employed as a semiconductor material for photocatalysis because of its appropriate band gap, excellent stability, robust oxidation capacity, cost-effectiveness, non-toxicity, and simple fabrication process [11]. However, the use of pristine ZnO in photocatalytic processes may lead to electron–hole recombination, which reduces degradation efficiency [12]. Several methods have been explored to enhance waste degradation efficiency, such as the addition of noble metals, the incorporation of other semiconductors, utilizing carbon nanotubes, and performing morphological modifications [12,13,14,15,16,17].

ZnO or ZnO-Ag composite materials can be synthesized using liquid and gas-phase methods. Liquid methods include sol–gel, spin coating, hydrothermal, solvothermal, coprecipitation, microwave-assisted synthesis, and chemical batch deposition [15,18,19,20,21]. These methods provide several benefits, such as the capability to be conducted at room temperature and atmospheric pressure, along with their relative simplicity. However, a common drawback is that the process tends to be time-consuming and requires solvents needing further treatment to be separated from the final product. In a prior study, ZnO was successfully produced using a simple process involving green synthesis with Cosmos Caudatus leaf extract [22]; however, the method’s lengthy steps and the non-uniform particle morphology were disadvantages. Gas-phase methods also offer promising techniques that do not require further separation to remove solvents, e.g., atomic layer deposition, physical and chemical vapor deposition, and electrospraying [23,24,25,26]. However, these methods have the drawback of requiring high temperatures and vacuum pressure. Kusdianto et al. (2019) examined how varying Ag concentrations influences ZnO formation through a gas-phase method in a flame reactor, their findings indicating that a Ag concentration of 5 wt% yielded the highest photocatalytic performance [27]. Their method is very simple, using LPG (fuel source) and air (oxidizer). However, maintaining stable temperatures in a flame reactor is quite challenging [28,29].

In previous studies [10,12], spray pyrolysis (SP) was employed as the synthesis method for ZnO-based photocatalysts under controlled temperature conditions using an electric heater, allowing precise temperature adjustments during synthesis. These studies investigated the effect of the nitrogen-to-oxygen carrier gas ratio during the spray pyrolysis synthesis on the properties of the resulting nanocomposites. The synthesized photocatalysts were subsequently applied for the photocatalytic degradation of dye pollutants in textile wastewater collected from the Gresik region of Indonesia. However, the degradation efficiency remained low, with results below 50%, and when pure oxygen was used, the maximum degradation efficiency reached only 25% within 90 min. A noted disadvantage of this method was the use of pure nitrogen and oxygen as carrier gases, which resulted in high operational costs. The novelty of this study lies in its comparative analysis of two particle synthesis methods utilizing aerosol technology, along with the adoption of oxygen as a carrier gas to reduce operational costs. Furthermore, computational fluid dynamics (CFD) simulations are employed to elucidate the underlying mechanisms occurring during the particle synthesis process. By comparing these two synthesis methods, the results will offer insight into which approach produces ZnO-Ag photocatalysts with properties most suitable for the photocatalytic degradation of organic pollutants.

## 2. Results and Discussion

**Figure 1:**
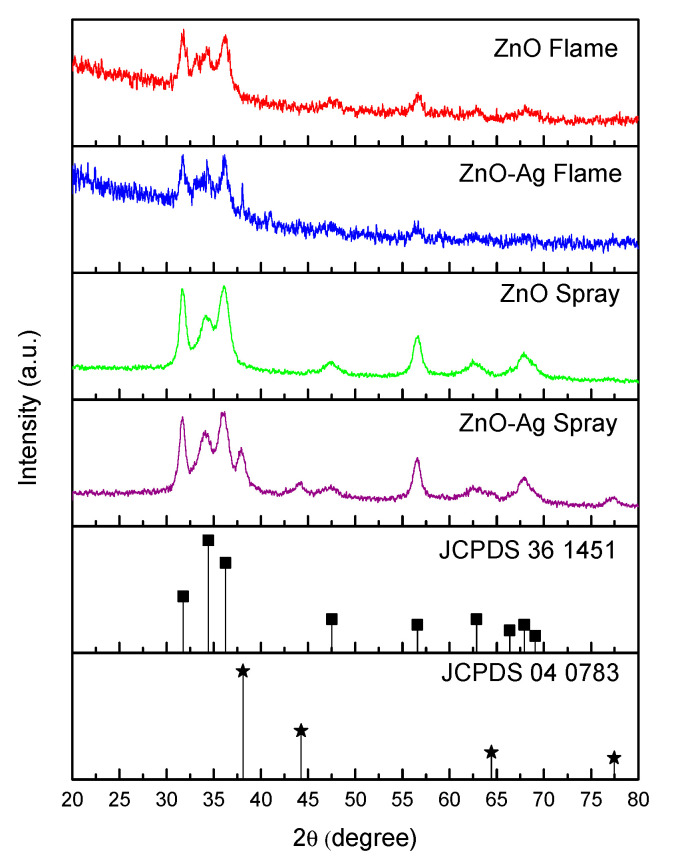
XRD spectra of ZnO material fabricated via spray pyrolysis and flame spray pyrolysis.

The XRD analysis results for ZnO and ZnO-Ag synthesized via flame and spray pyrolysis are shown in Figure 1. XRD spectra showed peaks at 31.6°, 34.3°, 36.3°, 47.6°, 56.7°, 62.9°, and 68°, corresponding to the crystal orientations (100), (002), (101), (102), (110), (103), and (112), respectively. According to JCPDS data, all the formed samples have a hexagonal Wurtzite (HW) crystal structure, which is the most stable structure, and this result is consistent with previous studies [12,27,30]. Additionally, the sharp and well-defined peaks with minimal broadening in the XRD spectra indicate that the produced particles exhibit high crystallinity [31,32]. It is observed that XRD spectra of particles synthesized via flame pyrolysis contain some significant noise, while those obtained from spray pyrolysis appear smoother. Furthermore, several additional peaks were clearly observed at 38°, 44.2°, and 77.4° in the ZnO-Ag nanocomposite synthesized through spray pyrolysis, corresponding to crystal orientations (111), (200), and (300), respectively. This supports the results of earlier studies by confirming that Ag is present in the nanocomposite. For the sample synthesized using flame pyrolysis, the strongest Ag peak was observed at 2*θ* = 38°.

The Scherrer equation was used to estimate the crystal diameter (*D*) of the produced particle based on the intensity of the highest peak in XRD spectra, as shown below:

(1) $$D=\frac{k\;\lambda }{B\cos ϴ}$$

*B* is the full-width at half maximum; *θ* is the peak angle employed; *λ* is the wavelength of the X-ray source, which has a value of 0.154 nm; and *k* is a constant with a value of 0.9. The Scherrer equation was applied using all observable ZnO diffraction peaks in the XRD spectra, including the (100), (002), (101), (102), (110), (103), and (112) planes, to estimate the crystallite sizes. The reported crystallite size represents the average value calculated from these peaks. The calculation results showed that the crystal diameter of ZnO produced via the flame method was slightly smaller compared to that via spray pyrolysis (Table 1); however, the difference is minimal, approximately 3 nm. The crystal diameter of the ZnO-Ag synthesized using both the flame and spray methods did not show significant variation. It can thus be concluded that the crystal size is neither affected by the method used nor the addition of Ag. This is consistent with previous studies stating that the crystal size is not influenced by Ag addition, although the presence of Ag lowers the phase transformation temperature of the crystal [5,33].

**Figure 2:**
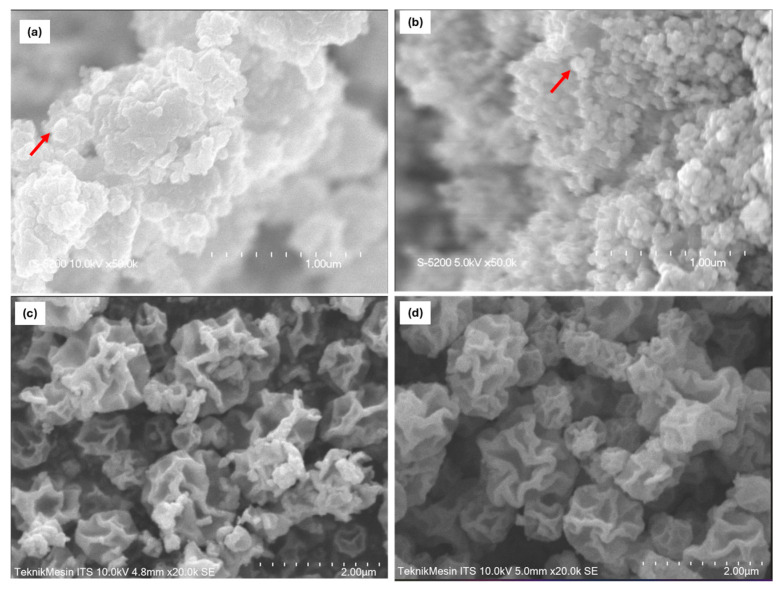
SEM images of ZnO particles fabricated using flame (**a**) and spray pyrolysis (**c**) methods. SEM images of ZnO-Ag 5%wt prepared by flame (**b**) and spray pyrolysis (**d**).

Figure 2 shows the morphology of the particles produced using both the flame (Figure 2a,b) and spray (Figure 2c,d) pyrolysis methods, examined through SEM analysis. In general, the particles produced via the flame spray pyrolysis method have a spherical shape and a smooth surface, as indicated by the red arrow in Figure 2. The particle diameter obtained via the flame synthesis (FS) is smaller than that via spray pyrolysis (SP), as described in Table 1. The ImageJ software program was used to measure several hundred particles from SEM pictures to estimate the average particle diameter. Based on the results, the average diameters for ZnO and ZnO-Ag synthesized using the flame method are 40 nm and 46 nm, respectively. Although the difference in average particle diameter is not significant, the morphology of ZnO without Ag doping has substantial agglomeration. With the addition of 5% Ag, the resulting particles show less agglomeration, which indicates that the addition of Ag can prevent agglomeration and inhibit particle growth, as also reported in previous studies [34]. The spray pyrolysis process produced particles with a highly wrinkled morphology, leading to a significantly increased pore diameter (18.6 nm for ZnO Spray and 9.5 nm for ZnO–Ag Spray) compared to the relatively spherical particles obtained via the flame method (4.5–5.7 nm). Despite having similar BET surface areas (237–284 m^2^/g), the larger pore sizes in spray-derived samples suggest enhanced accessibility for dye molecules and more efficient diffusion pathways during photocatalytic reactions.

This morphological difference plays a critical role in photocatalytic performance. The wrinkled structures not only increase the number of surface defects (which can act as reactive sites) but also reduce mass transfer resistance, facilitating more effective interaction between methylene blue and the ZnO surface. The improved degradation performance observed in spray-synthesized samples can therefore be attributed not merely to their surface area but also to their more favorable pore architecture and surface morphology.

In contrast to flame synthesis, the particles produced via spray pyrolysis do not have a spherical shape but tend to be wrinkled and porous and resemble flowers, as shown in Figure 2c,d. SEM scans show an average particle size of about 1.2 μm, which is significantly larger than that via the flame method. This difference is likely due to the distinct particle formation mechanisms between flame and spray pyrolysis. Furthermore, the Ag doping addition leads to a rise in particle size, consistent with the results of Dehimi et al. [35] and Kusdianto et al. [5]. The metal-induced crystallization phenomenon, which results in particle collisions and mergers, is responsible for this rise in particle diameter. Moreover, the heat transfer process among ZnO particles may become more pronounced in the presence of Ag nanoparticles.

**Figure 3:**
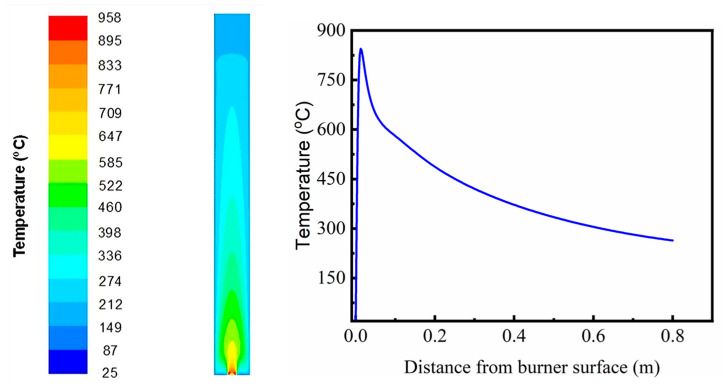
Temperature distribution of flame reactor using CFD simulation.

To explain the possible particle formation mechanism as described above, computational fluid dynamics was then used. The temperature distribution of flame reactor obtained from CFD simulation is shown in Figure 3. The simulation results show that the temperature distribution forms a vertical cone-shaped profile, suggesting stable diffusion combustion concentrated along the main axis. The axial temperature distribution shows that the gas temperature gradually decreases as the distance from the burner surface increases. The maximum temperature in the flame region reached 958 °C, located near the burner surface. The decrease in axial temperature can be attributed to heat energy dissipation from the reaction zone to the surroundings through convection and radiation mechanisms [36]. Additionally, the radial temperature distribution shows that the highest gas temperature remains focused in the center and decreases toward the domain walls. This indicates that heat transfer in the radial direction is relatively limited, as most of the heat is distributed axially, following the main flow direction. The simulation results show that the flame zone is divided into two regions, namely heating and cooling. The heating zone appears shorter than cooling, primarily contributing to the droplet evaporation process, while the cooling zone plays a role in the transformation from vapor to particle phase [37].

**Figure 4:**
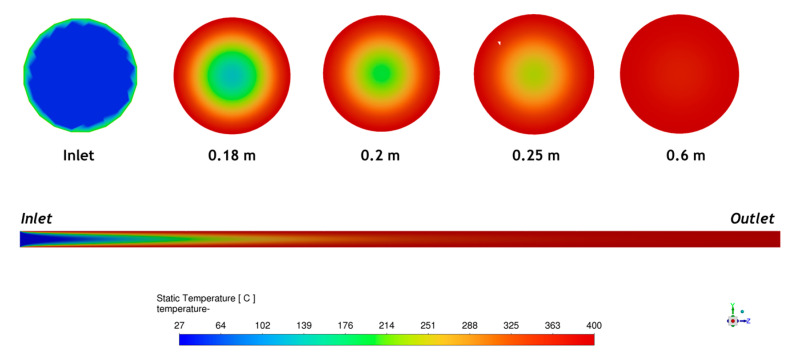
Static temperature contour along central axis of tubular furnace.

Figure 4 shows the static temperature distribution along the central axis of the tubular furnace during the spray pyrolysis process. The simulation was performed to analyze the temperature development as sprayed particles travel from the inlet toward the outlet. As shown in Figure 4, the temperature did not instantly reach the set target of 400 °C at the inlet, but the particle experienced a gradual heating process, forming a clear axial temperature profile. Initially, the region near the inlet had lower temperatures (represented by blue and green color gradients), indicating the entry of relatively cooler droplets or gas. As the flow proceeded downstream, the temperature gradually increased due to continuous heat transfer from the furnace walls to the moving particle and carrier gas, finally approaching the desired temperature of 400 °C near the outlet section (represented in red). The contour plot further validates this observation by showing a smooth and uniform axial thermal gradient. The temperature distribution is symmetric along the axis, indicating a well-controlled and stable thermal environment inside the reactor. The color mapping shows that the low-temperature zone (blue-green region) is confined near the inlet, and the high-temperature zone (red) dominates the outlet region.

Based on the results of the temperature profile simulation, the hypothesis regarding the particle formation mechanism is outlined as follows. The particle formation process in flame pyrolysis begins with the injection of precursor solution droplets into the flame zone, as previously reported by Mäkelä et al. [38]. CFD simulations confirm that the flame reactor consists of two distinct regions: a heating zone and a cooling zone. When the droplets enter the heating zone, where the maximum temperature can reach approximately 958 °C, the solvent within the droplets evaporates rapidly. This evaporation induces solute precipitation at the droplet surface, forming a solid shell layer before complete solvent evaporation occurs. The accumulation of vapor pressure inside this encapsulated droplet builds internal pressure, eventually leading to a micro-explosion, which breaks the droplet into smaller fragments. These fragments subsequently undergo further evaporation and thermal decomposition of the precursor to form ZnO. Upon entering the cooling zone and reaching a supersaturation state, nucleation of nanoparticles occurs, followed by surface growth, where the particles grow larger as more material condenses onto their surfaces. This is supported by SEM data, showing that the particle size from the flame process is approximately 40 nm.

In contrast to the flame process, spray pyrolysis may involve a different particle formation mechanism. The droplets generated via the nebulizer experience slow evaporation. Simulation results indicate that a uniform temperature of 400 °C is maintained in the spray reactor when the length is 0.6 m. As the droplets shrink due to evaporation, the precursor particle collides in the droplet. Once the decomposition temperature is reached, the precursor transforms into ZnO particles with a flower-like and non-uniform morphology. The decomposition temperature of zinc acetate is in range of 350–400 °C [22,39,40].

**Figure 5:**
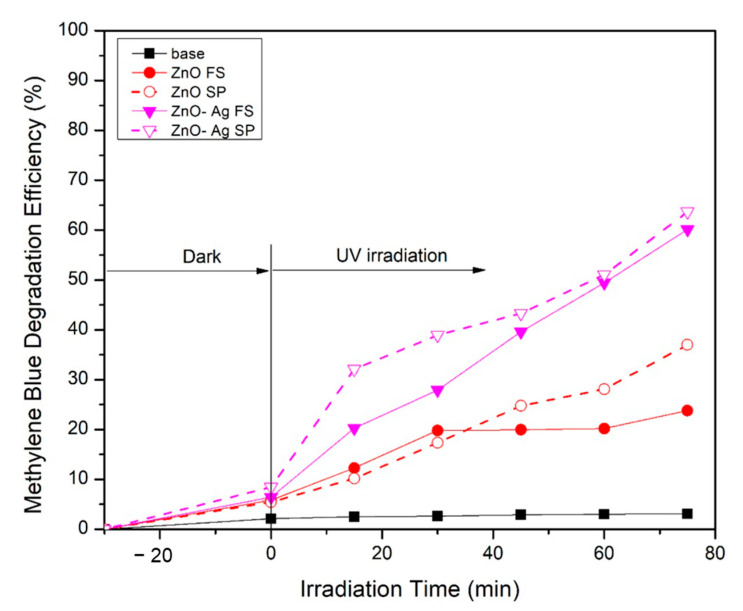
Photocatalytic activity under UV irradiation of ZnO and ZnO-Ag material fabricated using flame and spray pyrolysis methods under UV light irradiation.

Figure 5 shows the methylene blue degradation efficiency (MBDE) against irradiation time. The addition of Ag improved the degradation efficiency using both flame and spray pyrolysis methods. For the flame method, the degradation efficiency for pristine ZnO was around 22% and increased to 60% when 5% Ag was added. Ag prevents electron–hole recombination by acting as an electron acceptor. The photocatalytic mechanism starts immediately when the semiconductor material is exposed to photon energy. Upon exposure to photon energy equal to or greater than the band gap of ZnO, electrons are excited from the valence band to the conduction band [41]. The presence of Ag facilitates the transfer of electrons due to the Schottky barrier, effectively trapping them and preventing their recombination with holes. This results in a higher concentration of holes in the valence band, which interact with hydroxyl groups to produce hydroxyl radicals. These radicals play a crucial role in the degradation of methylene blue. In contrast, pristine ZnO lacks such an electron-trapping mechanism, leading to a higher probability of electron–hole recombination, reduced generation of hydroxyl radicals, and ultimately lower degradation efficiency. A similar trend was observed for samples synthesized via spray pyrolysis. The pristine ZnO achieved a maximum degradation efficiency of 38%, which increased to 64% upon Ag doping. These results are comparable to previously reported studies using different fabrication methods. For instance, Saadi et al. [42] achieved a maximum degradation efficiency of 72.3% for ZnO synthesized using the sol–gel method, while Maddu et al. [43] reported a 48% degradation efficiency using ZnO prepared by the hydrothermal method. Although the degradation efficiency of the spray pyrolysis method employed in this study reached 64%, slightly lower than the sol–gel result, it is important to highlight that spray pyrolysis offers better scalability, lower processing time, and simpler operational steps. Moreover, our work provides a unique insight into the droplet temperature distribution during synthesis, offering a more mechanistic understanding of particle formation and its effect on photocatalytic behavior. This aspect has not been extensively addressed in earlier publications.

MBDE, both for ZnO and ZnO-Ag, synthesized using the spray method was better compared to the flame method. Generally, many factors, including particle diameter, crystallite size, surface area, pore diameter, and particle shape, have a significant impact on photocatalytic activity. As previously explained, the difference in crystallite size is not significantly distinct. The influence of crystallite size can therefore be excluded as a factor contributing to the increase in degradation efficiency. Although the particle size produced via the spray method is approximately 30 times larger than that of the flame method, this particle size may not provide a dominant contribution. The effect of the surface area may also be ruled out as one of the parameters affecting the degradation efficiency.

**Figure 6:**
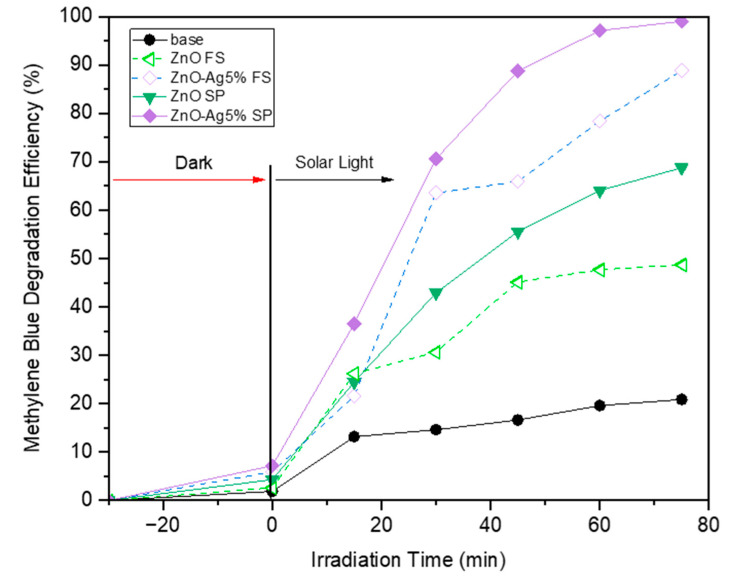
Photocatalytic activity of ZnO and ZnO-Ag materials fabricated using flame and spray pyrolysis methods under solar irradiation.

Additional experiments were conducted to evaluate the photocatalytic activity of the Zno and ZnO-Ag 5% particles under solar irradiation, aiming to simulate more realistic environmental conditions. As shown in Figure 6, both samples synthesized via flame and spray pyrolysis exhibited enhanced photocatalytic degradation of methylene blue under solar light compared to UV exposure. Remarkably, both flame and spray pyrolysis derived ZnO-Ag 5% achieved degradation efficiencies exceeding 95% within 75 min, while the pure ZnO particle degradation efficiency achieved 66 and 45% for spray and flame pyrolysis, respectively. This result is likely attributed to the localized surface plasmon resonance (LSPR) effect of silver nanoparticles, which enhances light absorption in the visible range and facilitates charge separation by acting as electron traps. The broader spectrum of solar light, which contains a significant portion of visible light, synergistically activates the Ag-doped ZnO, highlighting its superior activity under natural sunlight. This additional finding reinforces the practical applicability of the material for real-world, solar-driven photocatalytic applications.

**Figure 7:**
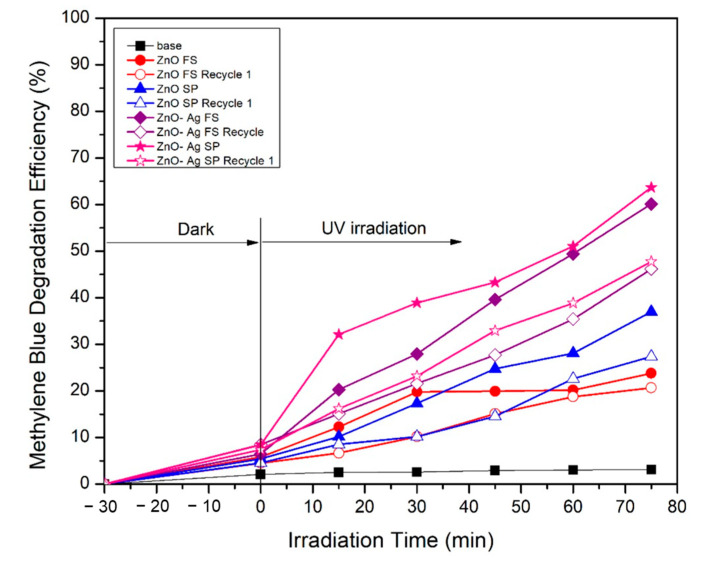
Reusability test of ZnO and ZnO-Ag 5%wt materials fabricated using flame and spray pyrolysis methods.

The reusability of photocatalysts is a key parameter in determining their practical applicability, especially in large-scale or long-term wastewater treatment applications. Figure 7 shows the photodegradation efficiency of ZnO and ZnO-Ag (5%) photocatalysts synthesized via both flame synthesis and spray pyrolysis over one-cycle under UV irradiation. All photocatalysts exhibited a gradual decline in activity upon repeated use which the decreasing of MBDE around 3–10%. This indicates the better structural integrity and surface activity of the synthesized material. The enhanced reusability performance of ZnO-Ag5% spray pyrolysis may be attributed to the uniform Ag dispersion and improved crystallinity facilitated by the spray pyrolysis method.

One of the factors that might enhance MBDE is morphology. In previous work, it was reported that photocatalytic activity can be increased by modifying the surface into nanorods or other structures [44,45]. Additionally, the pore diameter of particles synthesized using the spray method was 3–4 times larger than that of the flame method. This increased the level of contact between organic waste molecules (methylene blue/MB) and hydroxyl radicals, resulting in higher degradation efficiency. Although pristine ZnO has a larger pore diameter, the occurrence of electron–hole recombination leads to lower degradation efficiency compared to ZnO-Ag nanocomposite. These material characteristics, particularly those influencing photocatalytic performance such as morphology and pore structure, are inherently affected by the synthesis method employed. Understanding the practical implications of spray and flame pyrolysis methods is thus essential not only for optimizing material properties but also for evaluating their feasibility in real-world production. A comparison of their scalability, cost-efficiency, and industrial applicability provides deeper insights into how each method can be strategically leveraged depending on the intended application and production scale.

In terms of scalability and industrial applicability, both flame and spray pyrolysis offer distinct advantages and limitations. Spray pyrolysis provides better control over precursor delivery, droplet size, and deposition parameters, which translates to higher uniformity and reproducibility in particle morphology and composition. This makes it particularly suitable for producing doped nanoparticles with consistent quality, essential for applications requiring precise control over photocatalytic behavior. Moreover, spray pyrolysis is compatible with continuous and automated production setups, such as roll-to-roll coating systems and in-line thin-film deposition technologies, allowing seamless integration into large-scale industrial workflows.

On the other hand, flame pyrolysis is generally more cost-effective due to its simpler setup, lower energy requirements, and minimal equipment complexity. However, this simplicity often comes at the expense of limited control over particle size distribution and chemical homogeneity, which can affect photocatalytic efficiency. Despite this, flame pyrolysis remains attractive for large-scale synthesis where ultra-high purity or precise doping levels are not essential, such as in the bulk production of support materials or rapid prototyping of functional powders. Its high throughput and rapid synthesis time also make it ideal for mass production where moderate performance is acceptable.

In addition, the choice of carrier gas in spray pyrolysis significantly influences the process outcome. The use of oxygen as a carrier gas offers benefits such as enhanced combustion efficiency and improved oxidation of metal precursors, which can contribute to better crystallinity and phase purity of the resulting ZnO particles. However, its environmental and economic impacts should also be considered. Oxygen is generally more expensive than nitrogen or compressed air and demands more stringent safety systems due to its reactive nature. Although its use may lead to cleaner emissions, particularly by reducing nitrogen-based pollutants (e.g., NOx), oxygen production itself is energy-intensive and could increase the overall carbon footprint unless it is sourced from sustainable means. While oxygen may improve ZnO particle quality and photocatalytic performance, its large-scale application must therefore be carefully evaluated in terms of operational costs and environmental sustainability. As a compromise, a mixture of oxygen and nitrogen may offer a more balanced approach between performance benefits and ecological responsibility.

## 3. Materials and Methods

### 3.1. Materials

Zinc acetate dihydrate [Zn(CH_3_COO)_2_.2H_2_O], with 99.5% purity, and silver nitrate (AgNO_3_) served as precursors. Methylene blue (C_16_H_18_ClN_3_S) acted as a model for an organic pollutant. These chemicals were sourced from Merck (Darmstadt, Germany), with distilled water utilized as the solvent. All precursors were applied as received, without additional treatment. For the flame synthesis, liquefied petroleum gas (LPG, commercial grade, PT. Pertamina, Indonesia) was utilized as the fuel, while compressed air acted as the oxidizer.

### 3.2. Methods

The overall experimental procedures in this study can be categorized into two main stages: (1) precursor solution preparation and (2) material fabrication. In the first stage, precursor solutions containing zinc acetate and silver nitrate were prepared under aqueous conditions. In the second stage, these precursors were processed into ZnO and ZnO–Ag materials using two different fabrication approaches: flame spray pyrolysis and spray pyrolysis. Each fabrication method involved distinct thermal environments, yet both utilized the same ultrasonic nebulization system to generate fine droplets from the precursor solution.

#### 3.2.1. Precursor Solution Preparation

Zinc acetate dihydrate, a compound with high water solubility (~430 g/L at 20 °C), was selected as the primary zinc source. It was readily dissolved in deionized water using an ultrasonic bath without the need for heating or additional solvents, resulting in a 0.1 M zinc acetate solution. Silver nitrate was then added at a concentration of 5 wt% relative to the amount of zinc acetate, and the mixture was sonicated to ensure homogeneity. The prepared precursor solution was subsequently fed into an Omron ultrasonic nebulizer (NE-U780, Kyoto, Japan) operating at a frequency of 1.7 MHz. To maintain a consistent precursor volume and ensure stable droplet generation, fresh solution of the same composition was continuously supplied into the nebulizer at a controlled flow rate.

#### 3.2.2. Material Fabrication

**Flame Spray Pyrolysis**

The flame pyrolysis system used in this work was adapted from previous research [27] and constructed in-house. The droplets generated by the ultrasonic nebulizer were carried by compressed air into a flame reactor at a flow rate of 3 L/min. The carrier air was pre-filtered through silica gel to remove moisture. Liquefied petroleum gas (LPG) served as the fuel (0.3 L/min), while compressed air (2.8 L/min) acted as the oxidizer. Within the premixed flame reactor, the precursor droplets underwent rapid combustion and decomposition, forming ZnO or ZnO-Ag nanocomposites. The resulting particles were collected using an electrostatic precipitator (EP) maintained at 120 °C to prevent condensation. Any residual gases or particles not captured by the EP were directed to a water trap using a vacuum pump for final scrubbing and safe disposal.

**Spray Pyrolysis**

In the spray pyrolysis process, the same precursor solution and nebulization method were used. However, instead of a flame reactor, a horizontal tubular reactor heated electrically was employed. The reactor temperature was set and maintained at 400 °C using an integrated thermocouple and temperature controller. Similarly to the flame process, 3 L/min of compressed air was used to carry the aerosol droplets into the reactor. Inside the reactor, the solvent evaporated, and thermal decomposition of the precursors led to the formation of ZnO or ZnO-Ag nanocomposites. The products were collected by an EP powered by a DC high-voltage source. Exhaust gases and moisture were routed into a water trap for final filtration before being safely released. Further details of the setup can be found in references [10,12].

All collected particles were gathered in a powder collector and subsequently analyzed to determine their morphology, composition, and photocatalytic performance.

### 3.3. Numerical Simulations

To investigate the temperature distribution inside both flame reactor and spray tubular furnace, computational fluid dynamics (CFD) with ANSYS SpaceClaim 2024R2 was used. The governing equations, i.e., the conservation laws for mass, momentum, and energy, along with the equation of state, were solved for each mesh cell.

The flame reactor was modeled as a set of concentric stainless-steel cylinders with inner diameters of 16 mm (carrier gas), 19 mm (fuel gas), and 25 mm (oxidizer), each 1.5 mm thick and enclosed in an 800 mm Pyrex glass tube. The mesh consisted of 40,400 cells. Combustion was simulated using a single-step reaction of propane (C_3_H_8_) producing CO_2_ and H_2_O, with eddy dissipation used to model turbulence–chemistry interaction based on large-eddy mixing scales. The grid domain generated from meshing in the form of a rectangular mesh consists of 40,400 cells, 81,301 faces, and 40,902 nodes. In the combustion reaction simulation process, a single-step chemical reaction model was used, breaking down fuel gas (C_3_H_8_) into CO_2_ and H_2_O. Eddy dissipation was used to represent the turbulence–chemistry relationship. The large-eddy mixing scale served as the basis for the chemical reaction rate. Kinetic energy and dissipation rate were described by the flow turbulence model using the two-equation *k-ε* transport model, whose equations are formulated as follows [46]:

Equation of *k*:

(2) $$\frac{\partial }{\partial t}(\rho k)+\frac{\partial }{\partial xi}({\rho ku}_{i})=\frac{\partial }{{\partial x}_{j}}[(\mu +\frac{u_{t}}{\sigma_{k}})\frac{\partial k}{\partial x_{j}}]+G_{k}-\rho \varepsilon$$

Equation of *ε*:

(3) $$\frac{\partial }{\partial t}(\rho \varepsilon )+\frac{\partial }{{\partial x}_{i}}({\rho \varepsilon u}_{i})=\frac{\partial }{{\partial x}_{j}}[(\mu +\frac{\mu_{t}}{\sigma_{ℇ}})\frac{\partial ℇ}{\partial x_{j}}]+C_{1\varepsilon }(\frac{\varepsilon }{k})G_{k}-C_{2ℇ}\rho (\frac{\varepsilon^{2}}{k})$$

where *G_k_* is the turbulence energy production rate, while eddy viscosity is denoted as *μ_t_*, which accounts for mixing effects due to turbulence. The standard *k-ε* turbulence model uses a set of empirical parameters that have been extensively validated across various types of flow. The commonly used parameter values include *C*_1*ε*_ = 1.44, *C*_2*ε*_ = 1.92, *σ_k_* = 1.0, and *σ_ε_* = 1.3. Additionally, turbulent viscosity was calculated using the equation *μt = ρC_μ_k*^2^/*ε*, with a constant value of *C_μ_* = 0.09. The operating conditions for the combustion simulation process included a fuel gas flow rate of 0.3 L/min, a carrier gas flow rate of 3 L/min, and an oxidizer gas flow rate of 2.8 L/min.

The spray pyrolysis system was modeled as a horizontal tube (800 mm long, 20 mm inner diameter) with unidirectional flow from inlet to outlet. Meshing was performed using the Poly-Hexcore method in ANSYS Fluent to balance accuracy and computational cost. The final mesh contained 2550 cells with excellent quality: orthogonal quality ranged from 0.541 to 1.000 (mean 0.939), and skewness was low (min 1.31 × 10^−10^, mean 0.0587, max 0.459), indicating reliable simulation performance.

### 3.4. Characterizations

The crystal phase and crystallite diameter were ascertained using X-ray diffraction (XRD). Using a CuKα radiation source with a wavelength (*λ*) of 0.154 nm, XRD (Philips XPERT MFD, Amsterdam, The Netherlands) was run at an accelerating voltage of 40 kV and 30 mA. A sampling point of 0.02° and a scanning speed of 10°/min were employed. ZnO and ZnO-Ag formed via flame pyrolysis were examined using scanning electron microscopy (SEM, S-5200, Hitachi High Technologies, Tokyo, Japan) to determine their shape. Carbon-based ion sputtering was used to coat the samples prior to SEM testing. Using a SEM (FlexSEM1000, Hitachi High Technologies), the ZnO and ZnO-Ag morphology from spray pyrolysis was examined in the meantime. The ImageJ software program was then used to measure several hundred particles to estimate the particle size distribution. The surface area and pore diameter of the generated particle were analyzed using the nitrogen adsorption–desorption method (Autosorb-1, Quantachrome Instruments, Boynton Beach, FL, USA). Subsequently, the sample was degassed at a temperature of 150 °C before analysis. The BET surface area was calculated using adsorption data at a relative pressure (*P/Po*) ranging from 0.05 to 0.3.

To assess the photocatalytic performance, the synthesized ZnO and ZnO–Ag nanocomposite particles were subjected to methylene blue (MB) degradation tests under UV irradiation. A 250 ppm MB stock solution was prepared and diluted to 10 ppm for the tests, with calibration curves generated using a UV–Vis spectrophotometer (V-650, Jasco, Tokyo, Japan) at 665 nm. For each test, 0.05 g photocatalyst was dispersed in 60 mL of MB solution and stirred in the dark for 30 min to establish adsorption–desorption equilibrium. The photocatalytic tests were conducted in a custom-made, light-tight PVC box reactor equipped with a multi-stirrer system to ensure uniform mixing during irradiation. A 15 W UV lamp (Philips TUV, λ = 254 nm, ~1.2 mW/cm^2^ at solution surface) was placed 15 cm above the suspension inside the reactor. The reaction was performed at room temperature (27 ± 2 °C) under continuous stirring. At 15 min intervals, 3 mL aliquots were withdrawn, and then centrifuged at 1500 rpm for 10 min, and the supernatant was analyzed using UV–Vis spectroscopy to monitor MB concentration changes. The methylene blue degradation efficiency (MBDE) was calculated using

(4) $$MBDE\left[\%\right]=\frac{C_{0}-C_{t}}{C_{0}}\times 100\%$$

where *C*_0_ and *C_t_* are concentrations of MB before and after irradiation.

## 4. Conclusions

In conclusion, this study effectively synthesized ZnO and ZnO-Ag composites through both flame and spray pyrolysis methods. XRD analysis showed that the structure formed using both methods was hexagonal Wurtzite and that there was no significant difference in the crystal sizes produced, varying in the range of 3–6 nm. Particles produced using the flame method had a significantly smoother morphology and significantly smaller size, around 40 nm. In contrast, a rough morphology resembling flower-like structures was observed via spray pyrolysis, while the particle size was also 30 times larger than that synthesized using the flame method. Furthermore, the temperature profile obtained through computational fluid dynamics using ANSYS indicates that the flame exhibits a significantly higher temperature than the spray, resulting in smaller particles produced by the flame. Moreover, the addition of Ag particles caused an increase in the specific surface area, and the pore diameter of particles synthesized using spray pyrolysis was also three times larger than that of the flame method. Photocatalytic performance, evaluated using methylene blue degradation under UV and solar irradiation, showed that ZnO-Ag composites synthesized via spray pyrolysis achieved superior degradation efficiency, exceeding 95% under solar light. These results indicate the potential of spray-based ZnO-Ag photocatalysts for solar-driven environmental remediation.

## Figures and Tables

**Table 1:** Crystalline phase, crystallite size, average diameter, specific surface area, and pore diameter of ZnO and ZnO-Ag prepared via flame and spray pyrolysis.

Variable	Crystalline Phase ^a^	Crystallite Size ^b^ [nm]	Average Diameter ^c^ [nm]	Specific Surface Area ^d^ [m^2^/g]	Pore Diameter ^d^ [nm]
ZnO Flame	H. Wurtzite	3.3	40	237	4.5
ZnO-Ag Flame	H. Wurtzite	5.7	46	280	5.7
ZnO Spray	H. Wurtzite	6.5	1130	238	18.6
ZnO-Ag Spray	H. Wurtzite	5.8	1230	284	9.5

^a^ Crystalline phase was observed from XRD spectra based on JCPDS. ^b^ Crystallite size was calculated using Equation (1). ^c^ Average diameter was estimated from SEM data using ImageJ (version 1.54g). ^d^ Nitrogen adsorption–desorption method was employed to estimate the specific surface area and pore diameter.

## Data Availability

The original contributions presented in this study are included in the article. Further inquiries can be directed to the corresponding author(s).
